# Optimization of Heat Accumulation during Femtosecond Laser Drilling Borehole Matrices by Using a Simplex Algorithm

**DOI:** 10.3390/ma15144829

**Published:** 2022-07-11

**Authors:** Christian Lutz, Marcel Jung, Katrin Tschirpke, Cemal Esen, Ralf Hellmann

**Affiliations:** 1 Applied Laser and Photonics Group, University of Applied Sciences Aschaffenburg, Wuerzburger Strasse 45, 63743 Aschaffenburg, Germany; Jung-Marcel@gmx.de (M.J.); katrin.tschirpke@th-ab.de (K.T.); ralf.hellmann@th-ab.de (R.H.); 2 Applied Laser Technologies, Ruhr University Bochum, Universitaetsstrasse 150, 44801 Bochum, Germany; esen@lat.rub.de

**Keywords:** USP laser processing, laser drilling, optimization of heat accumulation, thermal simulation, optimization using a simplex algorithm

## Abstract

We report on an optimization study of percussion drilling thin metal sheets employing a high repetition rate, high power femtosecond laser with respect to the resulting heat accumulation. A specified simplex algorithm was employed to optimize the spatial drilling sequence, whereas a simplified thermal simulation using COMSOL was validated by comparing its results to the temperature measurements using an infrared camera. Optimization for drilling borehole matrices was aspired with respect to the generated temperature across the processed specimen, while the drilling strategy was altered in its spatial drilling sequence and by using multi-spot approaches generated by a spatial light modulator. As a result, we found that an optimization strategy based on limited consecutive holes in a Moore neighborhood led to reduced temperatures and the shortest process times.

## 1. Introduction

Ultra-short pulsed (USP) laser machining processes are, in general, associated with low thermal input into the processed work piece and thus a negligible heat affected zone, often referred to as cold ablation [1,2]. However, for high repetition rate lasers with high pulse energies and thus high average powers, pulse-to-pulse interactions may result in heat accumulation [3,4]. Such lasers have recently reached industrial-grade, allowing for efficient material processing such as drilling thousands of micro-holes per second [5,6,7,8]. This heat accumulation, in turn, results in an unexpected low quality of USP laser processing [9,10,11]. Particularly for thin materials, the accumulated heat may lead to the bending of the processed material, leading to shifting of the specimen out of focus. As a consequence (e.g., for laser drilling), this can result in a discontinuation of the process or unevenly drilled holes. To optimize drilling processes toward a higher quality of hole matrices, the systematic heat accumulation must be investigated. Therefore, we simulated the thermal process during drilling a large number of micro-holes in the laser single and multi-spot mode, and experimentally measured the charged heat by an infrared camera.

In particular, the size of the heat-affected zone, which results from a combination of laser parameters, the material properties, and the ablation process, is an important industry consideration for cutting and drilling applications using an USP laser. Aiming to increase the productivity of USP-processes, higher repetition rates and fluences are often used, which results in heat buildup and plasma shielding effects [12,13,14,15,16]. While heat accumulation generally results in negative effects, Gruner et al. [6] showed an increasing ablation rate for a high repetition rate of 48.78 MHz and a low fluence of 0.52 J/(cm^2^) during a drilling process without impairing the quality. These experiments indicate a decreasing influence of the applied fluence for very high repetition rates and a decreasing ablation threshold as a result of heat accumulation [6]. These findings are in agreement with Finger et al., who found an up to ten times higher ablation depth for increasing repetition rate, which was attributed to an increased energy transfer in combination with heat accumulation [4]. Bornschlegel et al. specifically investigated the heat formation during the USP-laser ablation of stainless steel 1.4301 by measuring the work piece temperature. By simulating the process with COMSOL Multiphysics, it was shown that the residual energy, which is below the ablation threshold of the Gaussian beam profile, does not contribute to the ablation process, but heats the surrounding material. In addition, the amount of residual energy that heats the surrounding material is shown to be larger the closer the applied fluence is to the ablation threshold of the material [17]. It can also be concluded that as long as the used fluence is similar to the calculated optimal range, which is around seven times the threshold fluence [18,19], a constant part of the heat input is an acceptable assumption. If the used fluence deviates significantly from this, non-uniform heating occurs during the process, so this assumption can no longer be made. Minor deviations can be explained by the plasma shielding effects due to the use of higher repetition rates [3,20,21]. Other authors have investigated the removal rate during a percussion drilling process due to the heat accumulation effects for different metals and laser parameters with a high power USP-laser with an average power of 300 W [22]. By doubling the pulse energy, the drilling time decreases two orders of magnitude, while a further increase only results in a slight reduction in drilling time, which can be explained by the particle lubrication effect [23]. Furthermore, in this study, the repetition rate showed no significant influence on the heat accumulation between 200 kHz and 800 kHz, which agreed with Zhao et al. [24]. Using pulse bursts, high ablation rates and a low surface roughness is possible [25]. Due to the high repetition rate, an increasing heat accumulation results in a higher absorption and rising ablation rate.

## 2. Experimental

We employed an Yb: YAG laser with an infrared emission at 1030 nm, output power of 100 W, and a variable repetition rate between a single shot and 40 MHz (Amplitude Tangor, Bordeaux, France). The laser was integrated into a micromachining system (RDX-1000, Pulsar Photonics, Herzogenrath, Germany) that used a galvo scanner (IntelliSCANse14, Scanlab, Pucheim, Germany) and an F-Theta lens (LINOS F-Theta Ronar, Qioptiq, Göttingen, Germany) with a focal length of 100 mm. The focal diameter $d_{o}$, measured by a high-resolution CCD camera (UI149xLE, IDS, Obersulm, Germany) was 41 µm ($1/e^{2}$). The resulting fluence was calculated according $\Phi =E_{p}/\left(\pi \cdot r_{0}^{2}\right)$, with $E_{p}$ being the pulse energy and $r_{0}$ the radius of the focal beam. Throughout the experiments explained in Section 4, a repetition rate of 50 kHz was used to drill the holes. A spatial light modulator (SLM)-based beam shaping module allows dynamic beam shaping to generate different spot distributions with a maximum frame rate of 60 Hz. To protect the LCOS-SLM (Hamamatsu X15223), an effective active liquid cooling system was integrated.

**Figure 1:**
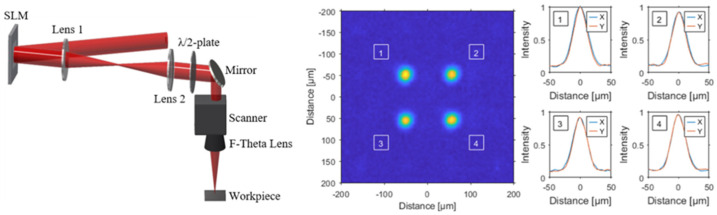
A schematic illustration of the setup for femtosecond laser ablation using a spatial light modulator (SLM) and a 4f-telescope with a galvo scanner including an F-Theta lens. Shown also are the measured intensity profiles of the four spots with a separation of 100 µm.

The drilling process was performed using both single spot and a multi-spot (2 × 2) beam splitter with a separation between the spots of 100 µm. The beam profile including the intensity distribution of the 2 × 2 spot profiles is shown in Figure 1. The required computer generated holograms were calculated using an iterative Fourier transform algorithm. After the initial calculation of the hologram, the uniformity of the single sub-beams in the focal plane was further improved by a feedback loop based weighted Gerchberg–Saxton algorithm [26]. Thus, a uniformity of 0.98 could be achieved for the four spot profiles shown in Figure 1.

To evaluate the drilled holes, a digital microscope (Leica DVM6A, Wetzlar, Germany) was used. The temperature measurements were conducted by an infrared camera (thermoIMAGER, Micro-Epsilon Messtechnik, Ortenburg, Germany) during the drilling process. The camera allows for imaging a temperature range between −20 °C and 1500 °C at a spatial resolution of 640 × 480 pixels with a maximum image recording rate of 32 Hz. As the low emission coefficient of stainless steel may lead to the falsification of the thermal measurement results, the steel sheet was thinly coated with a 7 µm-layer of a special camera lacquer in order to improve the quality of the temperature measurement by suppressing the ambient radiation.

Due to the frequent use of stainless steel such as for microfilters [27], injection nozzles [28], and surfaces with altered wettability properties [29], stainless steel foil (X5CrNi18-10) with a thickness of 50 µm was used in this study. For the thermal simulation, relevant thermo-physical properties were taken from literature (cf. Table 1). The reflectivity of the specific material on hand was determined experimentally for an angle of incidence of 90° in a spectral range between 190 nm and 2500 nm. The measurement setup consisted of a spectrometer (Spectro 320, Instrument Systems, München, Germany) with a spectral range from 190 nm to 2500 nm and a resolution of 0.1 nm. An equipped goniometer with an angular resolution of 0.1° was set with an angle of 90°, which corresponded to the machining situation in the drilling process. Furthermore, by using an integrating sphere, the measurement of the scattering sample was performed and measured via a detector port. For the applied laser wavelength of 1030 nm, the material showed a reflectivity of 67.5%.

## 3. Simulation

For the thermal simulation of the drilling process, the simulation software COMSOL Multiphysics was used with calculation steps of 0.005 s. To gain access to the temperature across the laser machined metal sheet on time scales of the machining process and data acquisition time of the thermal imager, we simulated the temperature evolution in the steady state (i.e., disregarding excitation and interaction effects on ultrashort time scales). As the infrared camera yielded the effectively generated temperature, a comparison to the simulated temperature in turn allowed us to deduce the correction factor for the simulation.

The defined drilling strategy was plotted based on two time-dependent functions in the x (holes per line) and y (holes per row) direction, with variables listed in Table 2. In Figure 3, the standard drilling sequence of a 5 × 5 array is schematically shown as an example. Furthermore, the sheet to be machined was simulated by using a convective heat flux $q_{L}$ in an air-filled environment, which was calculated by Equation (1).

(1) $$q_{L}=h_{L}\times \left(T-T_{ext}\right)$$

The heat flow was determined by multiplying the heat transfer coefficient $h_{L}$ of 6 $\frac{W}{m^{2}*K}$ with the difference between the ambient temperature $T_{ext}$ and the actual component temperature T.

The absorbed laser power was mapped over the interface of a heat transport in solids with a “Boundary Heat Source” node. The average power was calculated using the variables listed in Table 1 by Equation (2) and a drilling time of 0.02 s per hole.

(2) $$Q_{M}=m\times \left(c_{solid}\times \left(T_{m}-T_{ext}\right)+H_{m}+c_{liquid}\times \left(T_{v}-T_{m}\right)+H_{v}\right)$$

From the energy values calculated in this way, the power required per borehole for the simulation can be derived, taking into account the reflection coefficient. Based on the material properties from Table 1 and Equation (2), this led to a calculated required power of $Q_{0}=0.68\;W$. Due to losses in the drilling process caused by pulse-to-pulse interactions as well as plasma and particle shielding, the determined value in the experimental tests was not sufficient to produce a fully drilled through-hole. Experimental tests, in which the used pulse energy was slowly increased at pulse energy of 4 µJ, led to a through-hole in the material, which corresponded to an average power of $Q_{0}=0.8\;W$. This corrected value was used for the following tests as well as for the simulation. The resulting heat source of the simulation was calculated according to Equation (3). Here, the expression $gau\left(x,x_{00},r,y,y_{00}\right)$ represents the Gaussian beam profile and the term $rect\left(t_{L}\right)$ for the drilling time of a hole.

(3) $$q_{b}=\frac{2\times Q_{0}}{\pi r^{2}}\times \left(1-R_{c}\right)\times gau\left(x,x_{00},r,y,y_{00}\right)\times rect\left(t_{L}\right)\times 0.3$$

Bornschlegel et al. [18] investigated the heat accumulation in stainless steel during a USP-laser process for different repetition rates and fluence ranges by simulation and experiment. While using a fluence that is not in the range of the theoretically optimal fluence [31,32], a constant parameter can be used as a good approximation for the range of the theoretically optimal fluence. In order to represent a realistic heat input, Equation (3) was multiplied by the value 0.3, which was determined according to Bornschlegel et al. [20]. The function $gau\left(x,x_{00},r,y,y_{00}\right)$ represents the Gaussian beam profile and depends in this context on the start position (x,y), the current laser position ($x_{00},y_{00}$), and the spot radius r.

(4) $$gau\left(x,x_{00},r,y,y_{00}\right)=e^{\frac{{\left(x-x_{00}\right)}^{2}-{\left(y-y_{00}\right)}^{2}}{r^{2}}}$$

For the calculation of new drilling sequences, IBM ILOG CPLEX Optimization Studio (CPLEX) was used, which is a solver for linear and mixed integer problems based on the simplex algorithm. The accumulated heat of the drilling process is transformed into a thermal optimization problem based on the thermal simulation in COMSOL, which can be integrated and solved in CPLEX.

**Figure 2:**
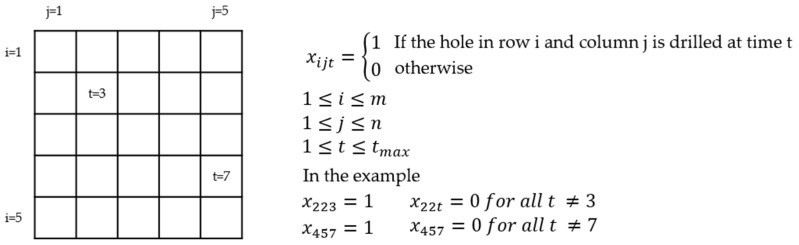
The schematic diagram for the explanation of the decision variables for the optimization problem.

The problem was modeled as an integer optimization problem with binary decision variables $x_{ijt}$ as depicted in Figure 2. The variable is one if the hole in row *i* and column *j* is drilled at time *t* and the variable is zero otherwise. As the objective, an artificial variable z is minimized with constraints, guaranteeing that the processing time is as short as possible. If $t_{max}$ is chosen too small, the optimization will not find a solution. A too large $t_{max}$ does not influence the optimal solution.

(5) $$\min\left(z\right)\mathrm{subject}\;\mathrm{to}\;t\cdot x_{ijt}\leq z\;\mathrm{for}\;\mathrm{all}\;i,j,t$$

The variable $x_{ijt}$ becomes one when a hole is drilled at time *t* and consequently *z* is greater than all times *t* at which holes are drilled. This objective minimizes the latest time and thus also minimizes the total processing time. Other constraints guarantee that each hole is drilled exactly once and that one position at most is drilled at any time *t*.

(6) $$\underset{t}{\sum }x_{ijt}=1\;\mathrm{for}\;\mathrm{all}\;\mathrm{positions}\;i,j$$

(7) $$\underset{i,j}{\sum }x_{ijt}\leq 1\;\mathrm{for}\;\mathrm{all}\;\mathrm{times}\;t$$

Several additional conditions were added to prevent heat accumulation in the metal sheet. For each 3 × 3 array, the number of possible holes drilled in time window [*t*, *t* + 8] was reduced from nine possible holes to a smaller number. The same procedure was carried out for each 5 × 5 field and each time window [*t*, *t* + 24], where theoretically, 25 holes would be possible. The allowed numbers become smaller with increasing time as the whole metal sheet becomes warmer.

The results of the optimization strategy were compared to a basic drilling strategy row by row with time breaks between every two consecutive holes, resulting in an identical total processing time.

## 4. Results and Discussion

**Figure 3:**
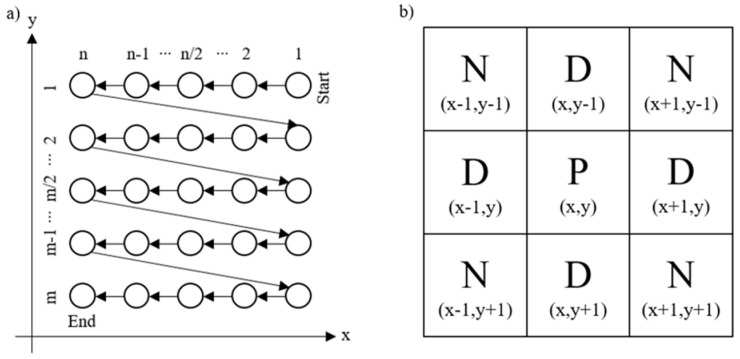
The schematic representation of the standard drilling sequence of a drilling array (**a**) and (**b**) the used Moore neighborhood relation around P with two horizontal and two vertical (D) and four diagonal neighbors (N).

**Figure 4:**
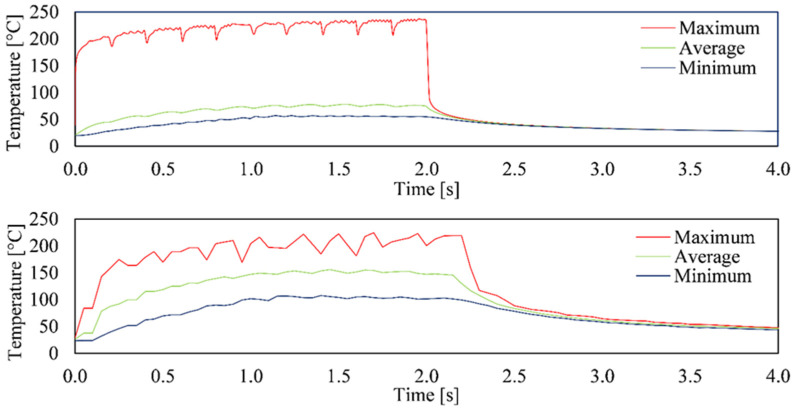
Comparison of the simulated (top) and experimentally (bottom) determined temperature curves for a 10 × 10 array with a separation of 100 µm. The holes were drilled with an average power of 0.8 W and a drilling time of 0.02 s.

To investigate the heat propagation into the material during the drilling process and to verify the heat simulation, in the first step, the original drilling sequence (Figure 3a) was simulated without optimization and carried out experimentally (Figure 4). In this sequence, drilling of a 10 × 10 array is conducted row by row, with a spacing of each hole of 100 µm. The temperature graphs resulting from the simulation and experiment are comparatively shown in Figure 4. Apparently both the attained temperature range of about 200 °C and the overall temperature profile in both the simulation and experiment were in good agreement. Both the periodic heating phases due to the drilling of each line and the subsequent jump as well as the cooling behavior to be expected according to Newton’s cooling law after the process could be clearly seen and were concordant. Furthermore, ten oscillations could be seen in the graph, each corresponding to the drilling of an entire row of the 10 × 10 array. The short drop in the maximum measured temperature in the area of the inflection point between the oscillations resulted from the jump from the last hole of a drilled row to the first hole of a new row. Please note that the finely displayed profile of the simulated temperature graphs resulted from the significantly smaller calculation steps compared to the frame rate of the thermal imaging camera. The high agreement of the results from the simulation and experiment allows for the validation of the drilling process so that this can be used to demonstrate the effects of changing the drilling sequence.

To reduce the temperature across the processed metal sheet by optimizing the drilling strategy, an approach from image processing was chosen [33,34]. The Moore neighborhood or figure-of-eight neighborhood considers the neighborhood relations of a defined pixel that is framed by other pixels in a square grid. The neighbors are all pixels that have at least one corner in common with the base area. Based on this principle, the whole array was now drilled in a box of nine (cf. Figure 3b), whereby a maximum of five holes could be drilled depending on the fulfillment of the sequence depicted in Figure 3b.

Here, the number of drilled holes allowed in each 3 × 3 array in time window [*t*, *t* + 8] was set to 5 at the beginning, 3 after time *t* = 11, and 2 after time *t* = 21. For each 5 × 5 array, the number was three times as large. As a result of the optimization strategy, there were times *t* when no hole was drilled and the process was interrupted by a waiting period.

Further drilling tests were carried out with the 2 × 2 beam splitter shown in Figure 1 due to a higher average power and thus a higher heat generation in the material. In addition, toward the benefit of responding to the industry’s demand for larger borehole arrays, this has the effect of the appearance of annealing colors, on the basis of which further evidence is provided regarding the temperatures generated in the material. A drill hole position calculated by the optimization software or the respective time interval represents here the simultaneous drilling of four holes of square arrangement.

**Figure 5:**
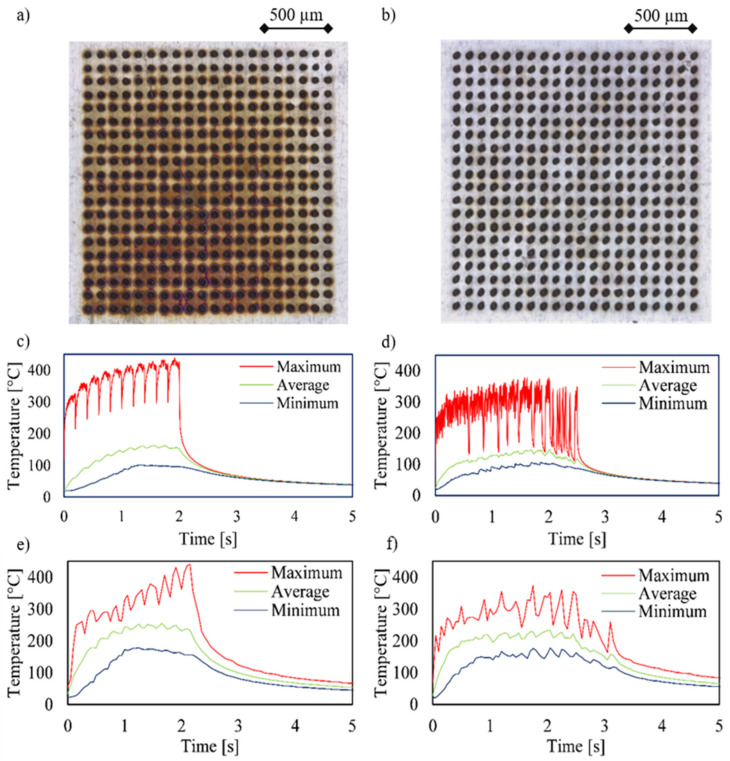
Microscope images (**a**,**b**), simulated temperature plots from COMSOL (**c**,**d**), and temperature plots (**e**,**f**) of a 20 × 20 array with 100 µm separation distance taken with a thermal imaging camera. The holes were drilled using a 2 × 2 beam splitter and a power of 2.3 W with a repetition rate of 60 kHz. (**a**,**c**,**e**) correspond to the standard drilling sequence, (**b**,**d**,**f**) to the optimized drilling sequence.

For further comparison and evaluation, data from the thermal simulations and experiments are shown for a 20 × 20 array drilled with a 2 × 2 beam splitter in Figure 5. The recorded image clearly shows the annealing colors through which the line-by-line standard drilling strategy can be seen directly. As the number of drilled lines increases, the heat accumulates in the material, so that the average value increases step by step, as can also be seen in the diagram. Looking at the image of the array from the upper right corner diagonally to the lower left corner, the steady increase in the temperature in the diagram reflects the increase in the tempering colors in the image. Thus, the peak of accumulated heat of slightly below 435 °C resulted from the most recently drilled holes. As above-mentioned in the explanation of Figure 4, ten oscillations were again visible in the graph, although 20 rows were drilled in this experiment. This resulted from the use of a 2 × 2 beam splitter, so two rows were always drilled simultaneously. When comparing the simulated graph and the graph taken with the thermal imaging camera, an identical trend as well as the same temperature range were observed. Please note the reduced measurement resolution due the sampling rate of the camera compared to the simulation. Due to the significantly smaller calculation steps of the COMSOL simulation (0.005 s) compared to the thermal imaging (0.05 s) and the limited spatial resolution of the camera, the detection of temperature values was not ideal. It can be assumed that this aspect as well as the small size of the boreholes partly led to an error in the recording of the temperature values. The average or minimum temperature showed a similar course, but deviated significantly with respect to the absolute temperature, which is an indication of the described deviations with respect to the resolution possibilities. The maximum temperature was not or only slightly affected by this since only the pixel with the highest temperature value was evaluated. The cooling behavior, which was shown by a steeply sloping curve in both the simulation and the experiment, also agreed well.

As can be seen in the diagrams, the maximum temperature could be reduced by about 75 °C to almost 360 °C using the optimized strategy. This and the significantly lower occurrence of annealing colors indicate a lower heat input in the component. When drilling with the optimized drilling strategy, there was also a constant increase in temperature up to the last hole, but this was much less pronounced. However, the behavior of a line-by-line increase in temperature was interrupted by the change in the drilling sequence. This resulted in the standard strategy with a decrease in the temperature after each drilled line, whereby, however, the temperature was higher with each line beginning than with the previous. This confirms the assumption that the material is not able to dissipate all the heat into the surrounding material and into the air after a line has been drilled, and this leads to heat accumulation. In addition to the optimized drilling strategy, the 25% longer process time caused by the break times could be another reason for the qualitatively better drilling result. In order to reflect this increased process time in the initial sequence, and thus to exclude any effects that may occur, a corresponding delay of 1 ms was inserted between the drilling of the individual holes.

**Figure 6:**
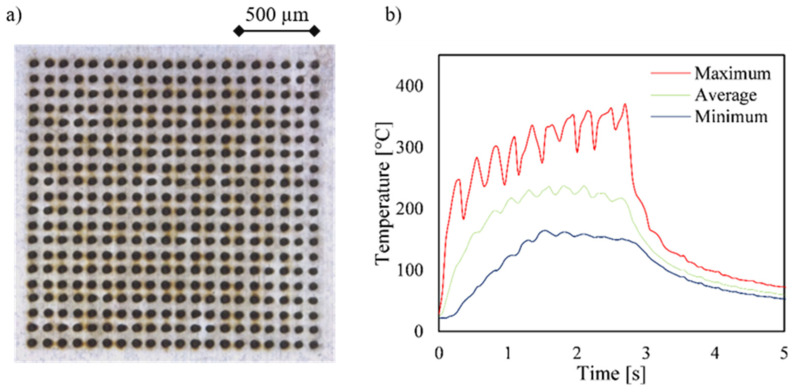
The microscope image (**a**) and the temperature plots (**b**) of a 20 × 20 array with 100 µm separation distance taken with a thermal imaging camera. The drilling process, with the optimized drilling sequence, using a 2 × 2 beam splitter and a power of 2.3 W with a repetition rate of 60 kHz was corrected with delays between the holes to increase the total process time by 25%.

The experimental results of the drilling test with the corrected process time by the inserted delay are shown in Figure 6. When comparing the two images, the qualitative result of the optimized drilling strategy was still the better one, which is shown by the lower annealing colors. However, it was noticeable that the duration of the process time and thus the short rest times between the drilling of the individual holes had a great influence on the thermal development, which was also confirmed by the temperature diagrams. Compared with the previous test (Figure 5), the maximum temperature dropped by about 40 °C, which explains the lower development of the annealing colors. Nevertheless, the optimized drilling strategy led to a 40 °C lower maximum temperature and a more uniform temperature gradient over the surface of the array. This shows that an optimized drilling strategy leads to an increase in quality with the same process time.

**Figure 7:**
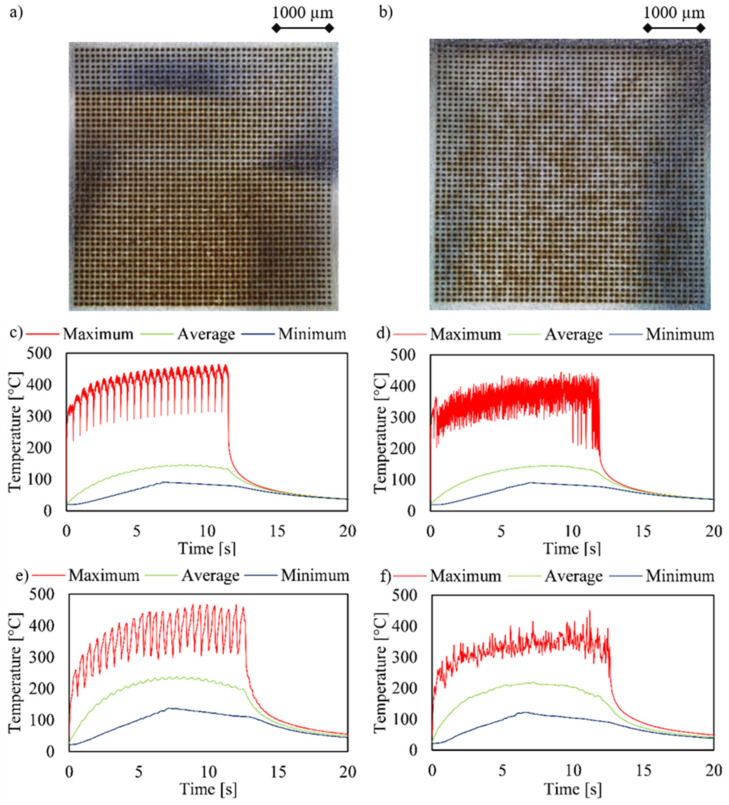
The microscope images (**a**,**b**), simulated temperature plots from COMSOL (**c**,**d**), and temperature plots (**e**,**f**) taken with a thermal imaging camera of a 48 × 48 array with a 100 µm separation distance. The holes were drilled using a 2 × 2 beam splitter and a power of 2.3 W and a repetition rate of 50 kHz. The drilling process was performed using the delays to correct the process time. (**a**,**c**,**e**) correspond to the standard drilling sequence, (**b**,**d**,**f**) to the optimized drilling sequence.

In the next step, the experiments were scaled up to a 48 × 48 array. The optimization of the drilling sequence was again calculated according to the Moore neighborhood. The obtained results, with the corrected process time by the inserted delay, which are shown in Figure 7, had similar characteristics as the results already shown in Figure 5. Again, the quality as indicated by the lower expression of annealing colors was higher and the temperature of the optimized strategy was lower compared to the standard drilling sequence. According to the 48 rows drilled in this experiment using a 2 × 2 beam splitter, 24 oscillations can be seen in the graph. However, it was noticeable that the proportion of annealing colors using the optimization had increased compared to the 20 × 20 array, which was also reflected in the lower difference in the maximum temperatures. The decrease in the effectiveness of the optimization was related to the fact that with larger arrays, a higher similarity to the standard strategy emerges. This results from the larger area and the associated property that there are only occasional wide jumps between successive boreholes and therefore the remaining boreholes are often drilled close together. On one hand, this results in fewer necessary break times and thus a shorter overall process time. The described trend correlates with the increasing component size, so that with a larger array, the advantage of optimization is smaller, but always evident.

## 5. Conclusions

Thermal simulations and a specified simplex algorithm were used to study and optimize the spatial drilling sequence of metal sheets using a high repetition, high power femtosecond laser in an up to 48 × 48-times multi-spot approach provided by a spatial light modulator. To increase the process efficiency while minimizing the generated temperature increase across the processed specimen, a Moore neighborhood was found for the drilling sequence to result in minimized accumulated temperatures with the inevitable being limited to a range of 45 °C to 75 °C, depending on the number of multi-spots.

## Figures and Tables

**Table 1:** The material properties of stainless steel 1.4301 (X5CrNi18-10) [30].

Material Property	Min Value	Max Value
Composition (in mass percent acc. DIN EN 10088-3)	C < 0.07, Cr = 17.5–19.5, Ni = 8–10.5, Mn < 2, Si < 1, *p* < 0.045, S < 0.03, N < 0.1	
Density ρ	$7850\;\frac{\mathrm{kg}}{m^{2}}$	$8060\;\frac{\mathrm{kg}}{m^{2}}$
$\mathrm{Specific}\;\mathrm{heat}\;\mathrm{capacity}\;c_{solid}$	$490\;\frac{J}{\mathrm{Kg}*K}$	$530\;\frac{J}{\mathrm{Kg}*K}$
$\mathrm{Specific}\;\mathrm{heat}\;\mathrm{capacity}\;c_{liquid}$	$649\;\frac{J}{\mathrm{Kg}*K}$	$689\;\frac{J}{\mathrm{Kg}*K}$
Thermal conductivity k	$14\;\frac{W}{m*K}$	$17\;\frac{W}{m*K}$
$\mathrm{Melting}\;\mathrm{temperature}\;T_{m}$	1400 °C	1450 °C
$\mathrm{Enthalpy}\;\mathrm{of}\;\mathrm{fusion}\;H_{m}$	$260\;\frac{\mathrm{kJ}}{\mathrm{kg}}$	$285\;\frac{\mathrm{kJ}}{\mathrm{kg}}$
$\mathrm{Evaporating}\;\mathrm{temperature}\;T_{v}$	3400 °C	3400 °C
$\mathrm{Enthalpy}\;\mathrm{of}\;\mathrm{evaporation}\;H_{v}$	$6500\;\frac{\mathrm{kJ}}{\mathrm{kg}}$	$6500\;\frac{\mathrm{kJ}}{\mathrm{kg}}$

**Table 2:** The parameters of the heat simulation in COMSOL Multiphysics of a 10 × 10 array.

Name	Expression	Value	Description
$T_{ext}$	293 [K]	293 [K]	Ambient temperature
$h_{L}$		$6\;\frac{W}{m^{2}*K}$	Heat transfer coefficient
n	10	10	Number of columns
m	10	10	Number of lines
$x_{0}$	(n−1)×d/2	0.45 [mm]	Laser position *x*-axis
$y_{0}$	(m−1)×d/2	0.45 [mm]	Laser position *y*-axis
r	20 [µm]	0.02 [mm]	Hole radius
$Q_{0}$	0.8 [W]	0.8 [W]	Average power
$R_{c}$	0.67	0.67	Coefficient of reflection
$t_{L}$	0.02 [s]	0.02 [s]	Drilling time per hole
